# AURKA/PLK1/CDC25C Axis as a Novel Therapeutic Target in INI1‐Deficient Epithelioid Sarcoma

**DOI:** 10.1111/cas.16438

**Published:** 2025-01-09

**Authors:** Akitomo Inoue, Hidetatsu Outani, Yoshinori Imura, Sho Nakai, Haruna Takami, Yuki Kotani, Hirokazu Mae, Seiji Okada

**Affiliations:** ^1^ Department of Orthopaedic Surgery Osaka University Graduate School of Medicine Osaka Japan

**Keywords:** alisertib, aurora kinase A, epithelioid sarcoma, polo‐like kinase 1, soft tissue sarcoma

## Abstract

Effective therapeutic strategies for epithelioid sarcoma (EpS), a high‐grade soft tissue sarcoma characterized by loss of integrase interactor 1 (INI1), have not yet been developed. The present study therefore investigated the association between INI1 loss and upregulation of the aurora kinase A (AURKA)/polo‐like kinase 1 (PLK1)/cell division cycle 25C (CDC25C) axis, as well as the therapeutic relevance of this axis in EpS. Notably, our findings showed that the reintroduction of INI1 in VA‐ES‐BJ cells significantly reduced proliferation, mitigated tumorigenicity, and negatively regulated the expression of AURKA and its downstream effectors, as well as the activation of PLK1 and CDC25C. These results suggest that INI1 deficiency enhanced EpS growth by upregulating the AURKA/PLK1/CDC25C axis. *AURKA* silencing using siRNAs inhibited VA‐ES‐BJ and Asra‐EPS cell proliferation by inactivating PLK1 and CDC25C. Alisertib, a selective AURKA inhibitor, exerted markedly greater antiproliferative effects on EpS cells than on normal human dermal fibroblasts, and these effects were dependent on INI1 deficiency. Inhibition of AURKA activity by alisertib induced G2/M cell cycle arrest and apoptosis via the inactivation of AURKA downstream effectors in EpS cells. Alisertib also significantly decreased VA‐ES‐BJ xenograft tumor growth. Taken together, our findings revealed that INI1 loss in EpS cells enhances the expression of AURKA and its downstream effectors and persistently activates PLK1 and CDC25C mediated by AURKA, making the cells reliant on the AURKA/PLK1/CDC25C axis. Therefore, the AURKA/PLK1/CDC25C axis activated by INI1 deficiency could serve as a novel therapeutic target for this devastating disease.

## Graphical abstract

This study confirmed that reintroducing INI1 in epithelioid sarcoma (EpS) markedly reduced cell proliferation, eradicated tumorigenicity, and downregulated AURKA and its downstream effectors, including PLK1 and CDC25C. Additionally, alisertib, a selective AURKA inhibitor, induced G2/M cell cycle arrest and apoptosis in EpS cells by inactivating AURKA's downstream effectors both *in vitro* and *in vivo*.

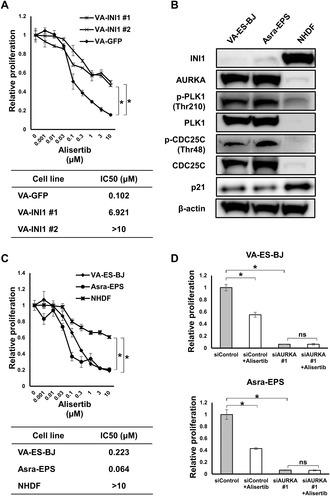

**Abbreviations**
AT/RT: atypical teratoid/rhabdoid tumor
AURKA: aurora kinase A
CDC25C: cell division cycle 25C
EpS: epithelioid sarcoma
EZH2: enhancer of zeste homolog 2
IC50: half‐maximal inhibitory concentration
INI1: integrase interactor 1
MRT: malignant rhabdoid tumor
NHDF: normal human dermal fibroblasts
PI: propidium iodide
PLK1: polo‐like kinase 1
RT‐qPCR: real‐time quantitative PCR
SMARCB1: SWI/SNF‐related matrix‐associated actin‐dependent regulator of chromatin subfamily B member 1
STS: soft tissue sarcoma
SWI/SNF: SWItch/sucrose non‐fermentable

## 1 Introduction

Epithelioid sarcoma (EpS), first described by Enzinger in 1970, is an aggressive soft tissue sarcoma (STS) that accounts for < 1% of all cases of STS [1, 2]. This malignant mesenchymal neoplasm exhibits epithelial differentiation and typically occurs in the extremities of young adults and adolescents. Given the general propensity of EpS for multifocal disease at presentation, local recurrence, and distant metastasis, affected individuals have shown unfavorable prognosis, with a 5‐year overall survival rate of 45%–70% [3, 4, 5, 6]. Although surgical resection has been the mainstay of treatment for localized EpS, options for patients with unresectable or metastatic disease remain undefined. Advanced EpS is quite challenging to manage given its limited response to chemotherapy, highlighting the urgent need for novel therapeutic approaches for patients with EpS.

The SWItch/Sucrose‐Non‐Fermentable (SWI/SNF) complex, also known as the BRG1/BRM‐associated factor complex, has been found to be involved in chromatin remodeling and transcriptional regulation, which contributes to cell differentiation and proliferation [7]. SWI/SNF‐related matrix‐associated actin‐dependent regulator of chromatin subfamily B member 1 (SMARCB1), otherwise known as integrase interactor 1 (INI1), is among the core subunit proteins in the SWI/SNF ATP‐dependent chromatin remodeling complex encoded at chromosome 22q11.2 [8]. Loss of tumor suppressor INI1 serves as a diagnostic criterion for malignant rhabdoid tumor (MRT) and atypical teratoid/rhabdoid tumor (AT/RT) [9]. Studies have also found that a majority of EpS cases present with loss of INI1 protein expression and biallelic *INI1* gene deletion [10, 11, 12, 13].

Aurora kinase A (AURKA), a member of the mitotic serine/threonine kinase family, is essential for several biological processes, including centrosome maturation and separation, spindle assembly, chromosome alignment, and G2 to M transition [14]. During mitosis, AURKA phosphorylates various substrates, including polo‐like kinase 1 (PLK1), which activates the nuclear localization of the cell division cycle 25C (CDC25C), and promotes entry into mitosis at the G2/M phase. Hence, AURKA overexpression has been implicated in tumorigenesis and associated with poor overall survival in patients with various cancers [15, 16, 17].

The loss of INI1 expression has been found to promote overexpression of AURKA, a direct downstream target of INI1, in MRT and AT/RT [18]. *SMARCB1* encodes the INI1 protein, which represses AURKA expression in specific cell types. Although *AURKA* knockdown induces mitotic arrest and apoptosis in RT cell lines, its role in INI1‐deficient EpS remains largely unexplored. Alisertib is a selective, potent, and reversible small‐molecule inhibitor of AURKA that has been studied in several cancers [19, 20, 21, 22, 23]. One previous report found a robust response following alisertib treatment in four children with recurrent AT/RT [24], whereas another phase II study investigated the outcomes of patients with MRT or AT/RT who received alisertib (SJATRT, NCT02114229) [25]. However, no data have been available regarding the efficacy of the pharmacological inhibition of AURKA for the treatment of EpS.

The present study explored whether the reintroduction of INI1 affects cell proliferation, tumorigenesis, and the expression of AURKA and its downstream effectors in INI1‐deficient EpS cell lines, VA‐ES‐BJ and Asra‐EPS. We also examined the role of AURKA/PLK1/CDC25C signaling in EpS cell lines. Finally, we investigated the antiproliferative effects of the selective AURKA inhibitor alisertib in EpS cells both *in vitro* and *in vivo*.

## 2 Materials and Methods

### 2.1 Cell Lines, Reagents, and Antibodies

This study used two human EpS cell lines, namely VA‐ES‐BJ and Asra‐EPS. VA‐ES‐BJ was purchased from the American Type Culture Collection, whereas Asra‐EPS was established at our laboratory [26]. Normal human dermal fibroblasts (NHDF) were obtained from Kurabo. Cells were grown in DMEM (Nacalai Tesque) supplemented with 10% FBS (Sigma‐Aldrich) and 1% antibiotics (100 IU/mL of penicillin and 100 μg/mL of streptomycin). Cells were cultured in a humidified atmosphere at 37°C and 5% CO_2_. Alisertib was purchased from AdooQ BioScience. All antibodies were commercially available and are summarized in Table S1. All cell lines underwent authentication through morphological examination, genotyping via PCR, and analysis of growth characteristics. Prior to experimentation, these cell lines were confirmed to be negative for *Mycoplasma* contamination through the use of the TaKaRa PCR Mycoplasma Detection Set (Takara Bio Inc.).

### 2.2 Cell Proliferation Assay

Cells were seeded into 96‐well plates at a density of 2 × 10^3^ cells/well. Cell proliferation was measured on a WST‐8 assay system using a Cell Count Reagent SF (Nacalai Tesque). The relative cell proliferation rate was calculated by measuring absorbances at 450 and 690 nm (reference wavelength) using a spectrophotometer.

### 2.3 Colony Formation Assay

A total of 500 cells were suspended in 1 mL of 0.5% SeaPlaque Agarose (Lonza) with normal growth medium and seeded into 6‐well Ultra‐Low cluster plates (Costar). The number of colonies (> 100 μm in diameter) was then counted under a light microscope 2 weeks later.

### 2.4 Western Blotting

For lysate preparation, cultured cells were washed with PBS and lysed in RIPA Lysis and Extraction Buffer (Thermo Fisher Scientific) supplemented with 1% protease/phosphatase inhibitor cocktail (Cell Signaling Technology). Tumor tissues were homogenized and lysed using a T‐PER Tissue Protein Extraction Reagent (Thermo Fisher Scientific). Protein concentrations were measured using bicinchoninic acid (Thermo Fisher Scientific). Thereafter, the cell lysates were separated using 4%–12% Bis‐Tris gels (Life Technologies) and then transferred to polyvinylidene difluoride membranes (Nippon Genetics). The membranes were incubated in TBS‐T containing 5% skim milk at room temperature and subsequently incubated with primary antibodies diluted in Can Get Signal Solution 1 (TOYOBO) at 4°C overnight and then with secondary antibodies diluted in Can Get Signal Solution 2 (TOYOBO) at room temperature for 1 h. After washing with TBS‐T, images were obtained using a ChemiDOC touch system (Bio‐Rad).

### 2.5 Real‐Time Quantitative PCR (RT‐qPCR)

Total RNA was extracted using an RNeasy Mini Kit (Qiagen) with gDNA Remover (TOYOBO) and reverse transcribed to cDNA using a ReverTra Ace qPCR RT Master Mix (TOYOBO). Gene expression was measured using a StepOnePlus Real‐Time PCR System (Applied Biosystems) and SYBR Green Real‐time PCR Master Mix (TOYOBO). Target gene expression levels were normalized to *GAPDH* levels. Relative expression was determined using the 2^−ΔΔCt^ method. PCR primers (forward and reverse) used in this study are detailed in Table S2.

### 2.6 RNA Interference

EpS cells were seeded into 6‐well plates at a density of 2 × 10^5^ cells/well and incubated overnight. Subsequently, the cells were transfected with 5 nM of siRNAs using Lipofectamine RNAiMax Transfection Reagent (Invitrogen). siRNAs targeting *AURKA, CDC25C*, and a non‐targeting negative control siRNA were purchased from Sigma‐Aldrich, while siRNA targeting *PLK1* and the corresponding negative control siRNA were procured from Cell Signaling Technology (Table S3).

### 2.7 Cell Cycle Analysis

EpS cells were seeded into 6‐well plates at a density of 4 × 10^5^ cells per well, incubated for 24 h, and then treated with alisertib or vehicle (DMSO) for 48 h. Thereafter, the cells were harvested and stained with propidium iodide (PI) solution (25 μg/mL of PI, 0.03% NP‐40, 0.02 mg/mL of RNase A, and 0.1% sodium citrate) for 30 min at room temperature. Cell cycle analysis was conducted using a BD FACSVerse flow cytometer (Becton Dickinson) and BD FACSuite software (Becton Dickinson).

### 2.8 Cell Apoptosis Assay

Cell apoptosis assay was performed using the MEBCYTO‐Apoptosis Kit. Each specimen comprised 4 × 10^5^ cells seeded into 6‐well plates overnight and subsequently incubated with alisertib or vehicle (DMSO) for 48 h. After harvesting the cells via trypsinization and washing with PBS, they were subsequently suspended in Annexin V staining buffer, stained with FITC‐Annexin V and PI for 15 min at room temperature, and analyzed utilizing a BD FACSVerse flow cytometer.

### 2.9 *In Vivo* Xenograft Models

Five‐week‐old athymic female BALB/c nu/nu nude mice were housed at the Institute of Experimental Animal Sciences, Osaka University Medical School, according to protocols approved by the Institutional Animal Care and Use Committee of the Osaka University Graduate School of Medicine. For the xenograft tumor growth assay, 1 × 10^7^ VA‐ES‐BJ cells were injected subcutaneously into the left side of the back. Therapy was initiated after the tumor reached an average size of 200 mm^3^. Mice were orally administered 30 mg/kg of alisertib or an equal volume of vehicle once daily, with five mice each allocated to the control and treatment groups. The composition of the solvent is 5% DMSO, 30% PEG 400, 5% Tween 80, and 60% double‐distilled water. Tumor volume and mouse body weight were measured every other day. Tumor volume was measured using a caliper and calculated according to the following formula: size = (length × width^2^)/2. Xenografted tumors were dissected and weighed after the tumor burden reached a maximum size of 2000 mm^3^. Tumor protein lysates were also harvested.

### 2.10 Immunohistochemistry

Tumor specimens were fixed in 10% neutral‐buffered formalin, embedded in paraffin, and cut into 4‐μm‐thick sections. Paraffin‐embedded sections were deparaffinized and dehydrated. Antigens were retrieved at 95°C for 10 min in 10 mM citrate buffer (pH 6.0). After blocking endogenous peroxidase activity with methanol containing 3% H_2_O_2_ for 10 min, the sections were reacted for 1 h with TBS containing 2% BSA at room temperature and then incubated with primary antibodies at 4°C overnight. Thereafter, the sections were incubated for 1 h with HRP‐conjugated secondary antibodies and stained with 3,3′‐diaminobenzidine tetrahydrochloride (Dako). Finally, the sections were counterstained using hematoxylin.

### 2.11 Statistical Analysis

All experiments were performed in triplicate, and all data were expressed as mean ± standard deviation. Statistical analyses were performed using Student's *t*‐test, with *p* < 0.01 indicating statistical significance.

## 3 Results

### 3.1 Reintroduction of INI1 Inhibited Cell Growth and Downregulated the AURKA/PLK1/CDC25C Axis in EpS Cells

**FIGURE 1:**
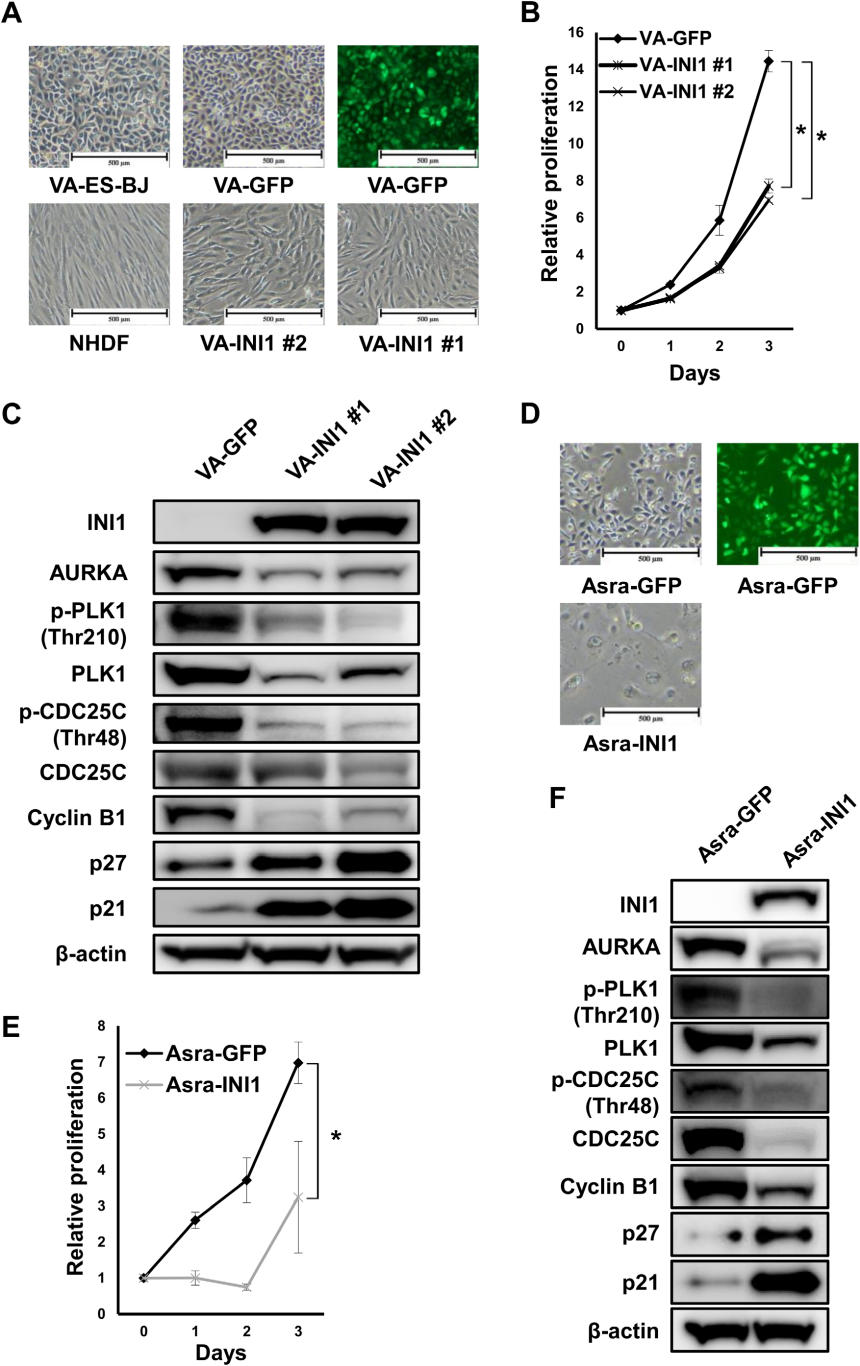
Reintroduction of INI1 inhibited proliferation of EpS cells and deactivated the AURKA/PLK1/CDC25C axis. (A) Cell morphology of VA‐ES‐BJ, VA‐GFP, VA‐INI1 (#1, 2), and NHDF cells. Scale bars, 500 μm. (B) Growth curve for VA‐GFP and VA‐INI1 (#1, 2) cells. Points, mean; bars, SD. *p < 0.01. (C) Expression of INI1 and AURKA‐related proteins in VA‐GFP and VA‐INI1 (#1, 2) cells. (D) Cell morphology of Asra‐GFP and Asra‐INI1. Scale bars, 500 μm. (E) Growth curve for Asra‐GFP and Asra‐INI1 cells. Points, mean; bars, SD. *p < 0.01. (F) Expression of INI1 and AURKA‐related proteins in Asra‐GFP and Asra‐INI1 cells.

To determine the relevance of INI1 loss in the VA‐ES‐BJ cell line, VA‐GFP and VA‐INI1 (#1, 2) clones were generated by overexpressing GFP and INI1 into VA‐ES‐BJ cells via lentiviral infection, respectively (Data S1). VA‐GFP cells displayed an ovoid and polygonal epithelial appearance similar to VA‐ES‐BJ, whereas VA‐INI1 cells were elongated and spindle‐shaped resembling NHDF (Figure 1A). VA‐GFP and VA‐INI1 clones were then analyzed for cell proliferation, colony formation, in vivo tumorigenesis, and protein expression. Impairment of cell proliferation was significantly greater in VA‐INI1 cells than in VA‐GFP cells (Figure 1B). Soft agar colony formation assay revealed that VA‐GFP cells showed a high colony‐forming capacity similar to VA‐ES‐BJ, whereas VA‐INI1 cells produced no colony (Figure S1A). Furthermore, the injection of 1 × 10^5^ VA‐GFP cells promoted the growth of xenografted tumors, whereas the injection of 1 × 10^7^ VA‐INI1 cells produced no tumor (data not shown). These results showed that the reintroduction of INI1 repressed proliferation and eliminated the colony‐forming and tumorigenic capacities of INI1‐deficient VA‐ES‐BJ cells.

Next, to ascertain whether AURKA and its downstream effectors (e.g., PLK1 and CDC25C) are affected by the reintroduction of INI1 in VA‐ES‐BJ cells, western blotting analyses were conducted on VA‐GFP and VA‐INI1 cells. AURKA is responsible for the Thr210 phosphorylation of PLK1, an essential mitotic kinase regulating multiple aspects of the cell division process [27]. In the presence of DNA damage, CDC25C is strictly localized to the cytoplasm through constant Ser216 phosphorylation to prevent premature mitosis entry [28, 29]. The inhibitory Ser216 residue becomes inactive during interphase, while the activating phosphorylation site Thr48 is constantly phosphorylated, potentially causing aberrant CDC25C activation. As shown in Figure 1D, VA‐INI cells showed lower protein expression levels of AURKA, PLK1, p‐PLK1 (Thr210), CDC25C, p‐CDC25C (Thr48), and cyclin B1 and higher expression levels of senescence‐associated markers, including p27 and p21, compared with VA‐GFP cells (Figure 1C). Comparable results were observed for Asra‐EPS (Figures 1D–F and S1B). Collectively, the reintroduction of INI1 into EpS cells reduced the expression of AURKA and its downstream effectors and induced senescence, indicating that loss of INI1 upregulates the AURKA/PLK1/CDC25C axis and inhibits senescence in EpS.

### 3.2 Silencing of *AURKA* Inhibited EpS Cell Proliferation by Inactivating AURKA/PLK1/CDC25C Signaling

**FIGURE 2:**
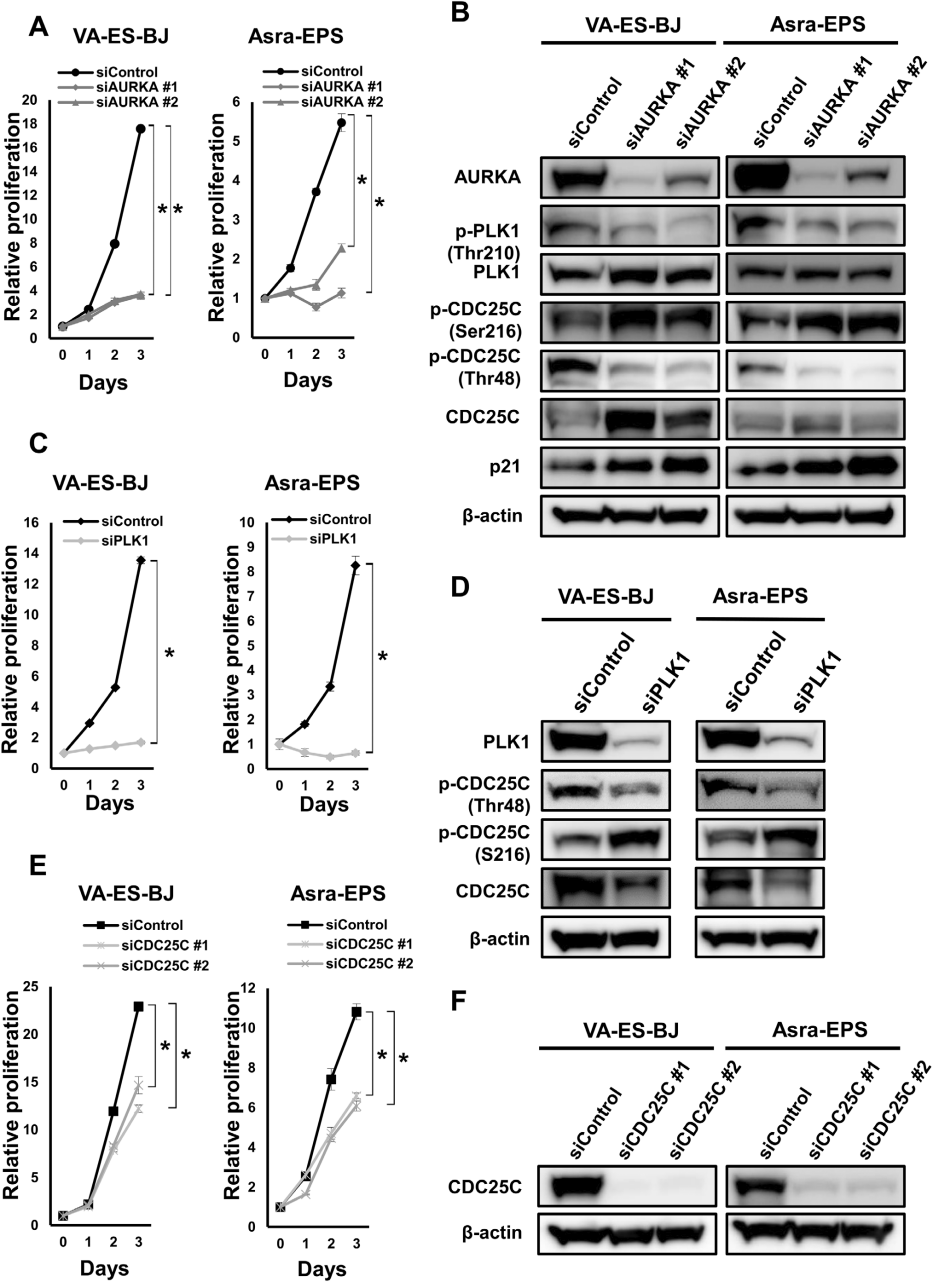
Silencing of *AURKA* decreased EpS cell proliferation by inactivating PLK1/CDC25C. (A) Growth curve for EpS cells transfected with anti‐*AURKA* siRNAs (#1, 2) or control siRNA. Points, mean; bars, SD. *p < 0.01. (B) Expression of AURKA‐related proteins in EpS cells transfected with anti‐*AURKA* siRNAs (#1, 2) or control siRNA. (C) Growth curve for EpS cells transfected with anti‐*PLK1* siRNA or control siRNA. Points, mean; bars, SD. *p < 0.01. (D) Expression of PLK1‐downstream proteins in EpS cells transfected with anti‐*PLK1* siRNA or control siRNA. (E) Growth curve for EpS cells transfected with anti‐*CDC25C* siRNAs (#1, 2) or control siRNA. Points, mean; bars, SD. *p < 0.01. (F) Expression of CDC25C proteins in EpS cells transfected with anti‐*CDC25C* siRNAs (#1, 2) or control siRNA.

To determine whether AURKA/PLK1/CDC25C signaling affects the proliferation of EpS cell lines, two types of anti‐*AURKA*‐specific siRNAs (#1, 2) were transfected into VA‐ES‐BJ and Asra‐EPS cells. Sufficient knockdown of *AURKA* with these siRNAs was confirmed through RT‐qPCR (Figure S2A). Notably, silencing of *AURKA* expression significantly inhibited the proliferation of VA‐ES‐BJ and Asra‐EPS cells (Figure 2A). *AURKA* inhibition with siRNAs decreased the protein levels of AURKA, p‐PLK1 (Thr210), and p‐CDC25C (Thr48) and increased the levels of p‐CDC25C (Ser216) and p21 (Figure 2B). To investigate the AURKA/PLK1/CDC25C axis, we also assessed the interaction between *PLK1* and *CDC25C* using siRNAs. In addition to *AURKA* knockdown, the silencing of these genes significantly affected proliferative capacity, and it was observed that *PLK1* silencing diminished the activity of its downstream target, CDC25C (Figure 2C–F). Additionally, we found that silencing of *AURKA* also inhibited colony formation (Figure S2B). These results suggest that silencing of *AURKA* inhibited the proliferation of EpS cells by inactivating the AURKA/PLK1/CDC25C axis.

### 3.3 EpS Cells With AURKA/PLK1/CDC25C Axis Hyperactivation due to INI1 Loss Were Highly Sensitive to Alisertib

**FIGURE 3:**
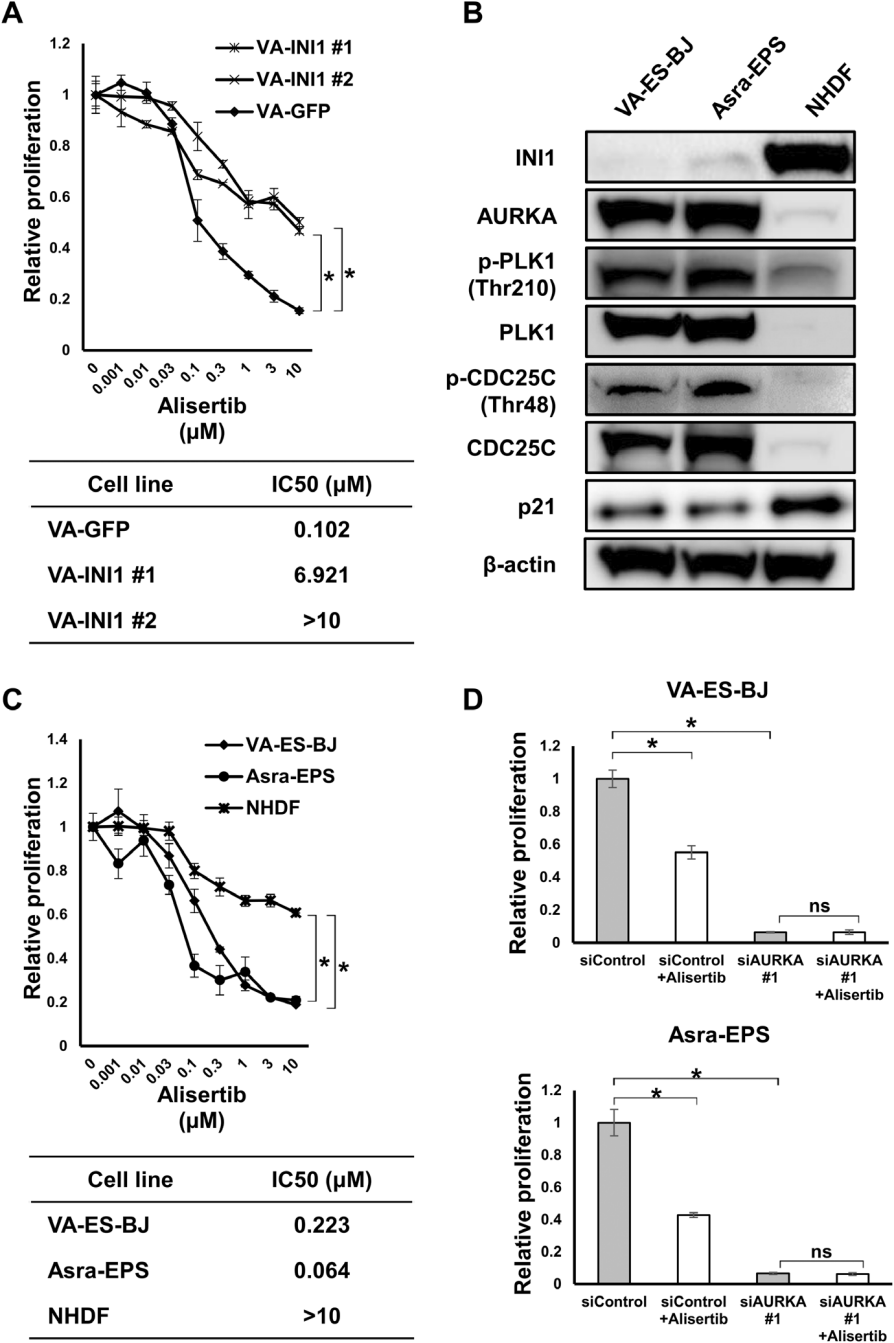
Alisertib exerted antiproliferative effects against INI1‐deficient EpS cell lines by inhibiting the AURKA/PLK1/CDC25C axis. (A) Sensitivity of VA‐GFP and VA‐INI1 (#1, 2) cells to alisertib. Cells were treated with various concentrations of alisertib or vehicle for 48 h, with their IC50 values being shown. Points, mean; bars, SD. *p < 0.01. (B) Expression of INI1 and AURKA‐related proteins in EpS and NHDF cells. (C) Sensitivity of EpS and NHDF cells to alisertib. Cells were treated with various concentrations of alisertib or vehicle for 48 h, with their IC50 values being shown. Points, mean; bars, SD. *p < 0.01. (D) Sensitivity of EpS cells transfected with anti‐*AURKA* siRNA (#1) or control siRNA to alisertib. Cells were treated with 0.3 μM of alisertib or vehicle for 48 h. Columns, mean; bars, SD. *p < 0.01.

We hypothesized that the AURKA/PLK1/CDC25C axis could be a therapeutic target for INI1‐deficient EpS. To ascertain whether the antiproliferative effects of alisertib, an oral selective small‐molecule inhibitor of AURKA, was dependent on INI1 deficiency in VA‐ES‐BJ, the relative proliferation of VA‐GFP and VA‐INI1 cells after exposure to various alisertib concentrations was assessed using the WST‐8 assay. Accordingly, we found that alisertib markedly inhibited VA‐GFP cell proliferation in a dose‐dependent manner, with a half‐maximal inhibitory concentration (IC50) value of 0.102 μM, but had limited antiproliferative effects on VA‐INI1 #1 and VA‐INI1 #2 cells, with IC50 values of 6.921 and > 10 μM, respectively (Figure 3A). Comparable outcomes were noted between Asra‐GFP and Asra‐INI1 (Figure S3A). These results demonstrate that targeting AURKA with alisertib disrupts the viability of INI1‐deficient EpS cells and that the reintroduction of INI1 into EpS cells reverses the inhibitory effects of alisertib.

Next, we investigated the activation of the AURKA/PLK1/CDC25C axis in two EpS cell lines, namely VA‐ES‐BJ and Asra‐EPS, and NHDF for comparison via western blotting. Notably, loss of INI1 expression was observed in VA‐ES‐BJ and Asra‐EPS cells, as previously described [30]. Both EpS cell lines showed higher levels of AURKA, PLK1, p‐PLK1 (Thr210), CDC25C, and p‐CDC25C (Thr48) and lower level of p21 than NHDF (Figure 3B).

We then examined the cell proliferation rates of the two EpS cell lines and NHDF using the WST‐8 assay to evaluate the antiproliferative effects of alisertib in EpS. Accordingly, we found that VA‐ES‐BJ and Asra‐EPS cells were more sensitive to alisertib in a dose‐dependent manner than NHDF cells (Figure 3C). The IC50 values of VA‐ES‐BJ, Asra‐EPS, and NHDF were 0.223, 0.064, and > 10 μM, respectively. These findings indicate that INI1‐deficient EpS cells in which AURKA/PLK1/CDC25C signaling was upregulated were more sensitive to alisertib than NHDF cells. We also assessed the impact of alisertib on colony formation and found that its administration markedly inhibited colony formation (Figure S3B).

To examine the inhibitory effects of alisertib on the phosphorylation of AURKA, AURKB, and AURKC, VA‐ES‐BJ cells were synchronized with nocodazole and then treated with various concentrations of alisertib. Western blotting analysis showed that lower concentrations of alisertib were required to inhibit AURKA phosphorylation at Thr288 than to inhibit AURKB or AURKC phosphorylation, indicating that alisertib has much higher selectivity for AURKA than for AURKB or AURKC (Figure S4).

Furthermore, to determine whether the effects of alisertib on EpS cell viability were specific to AURKA, VA‐ES‐BJ and Asra‐EPS cells transfected with anti‐*AURKA*‐specific siRNA (#1) or non‐targeting siRNA were treated with 0.3 μM of alisertib. As shown in Figure 3D, alisertib treatment significantly reduced the viability of EpS cells transfected with non‐targeting siRNA but not those transfected with siRNAs targeting *AURKA*. Taken together, silencing of *AURKA* attenuated the antiproliferative effects of alisertib in EpS cells, suggesting that alisertib treatment is specific for AURKA activity.

### 3.4 Alisertib Induced G2/M Cell Cycle Arrest and Apoptosis in EpS Cells

**FIGURE 4:**
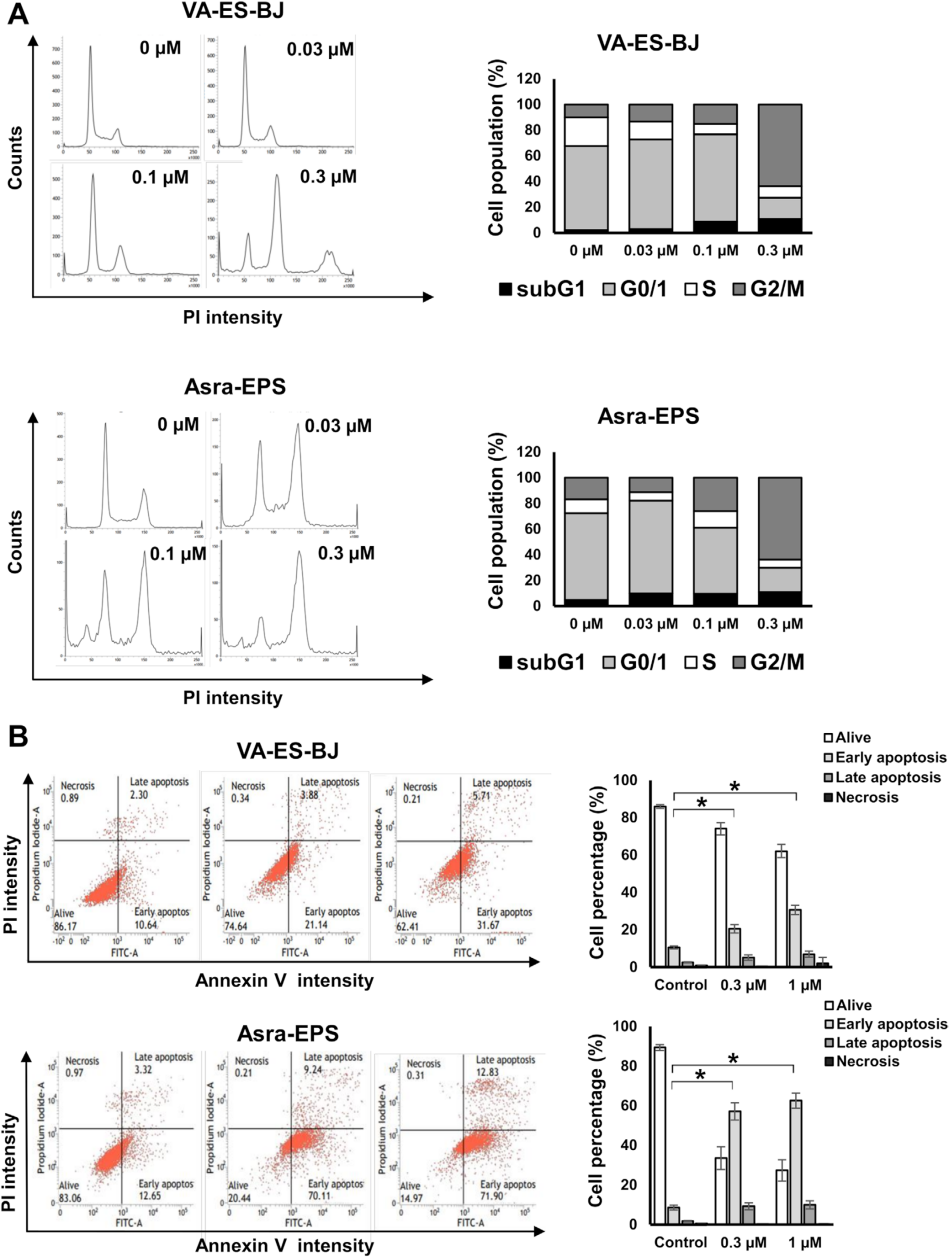
Alisertib induced G2/M cell cycle arrest and apoptosis in EpS. (A) PI staining fluorescence‐activated cell sorting analysis of the DNA contents of EpS cells in response to alisertib. EpS cells were treated with 0.03–0.3 μM of alisertib or vehicle for 48 h. The population of cells (%) at the subG1, G0/G1, S, and G2/M phases are shown. (B) Flow cytometry analysis of alisertib‐induced cell apoptosis. EpS cells were treated with 0.3 and 1 μM of alisertib or vehicle for 48 h. The percentages of viable, early apoptotic, and late apoptotic cells are shown. Columns, mean; bars, SD. *p < 0.01.

We subsequently evaluated the antiproliferative effects of alisertib on cell cycle distribution using a flow cytometer. The cell populations at the G2/M phase dose‐dependently increased in both EpS cell lines (Figure 4A). Moreover, this effect was accompanied by an increase in subG1 populations in both EpS cell lines, which was more pronounced in Asra‐EPS than in VA‐ES‐BJ (Figure 4A). These results indicate that alisertib treatment resulted in G2/M cell cycle arrest and apoptosis in EpS.

FITC‐Annexin V apoptosis was also evaluated via flow cytometry to determine the percentage of apoptotic cells. Notably, we found that the percentage of apoptotic cells also increased dose‐dependently in both EpS cell lines (Figure 4B). Consistent with the aforementioned results, lower concentrations were required to increase the percentage of apoptotic cells in Asra‐EPS than in VA‐ES‐BJ.

### 3.5 Alisertib Downmodulated AURKA/PLK1/CDC25C Axis in EpS Cells

**FIGURE 5:**
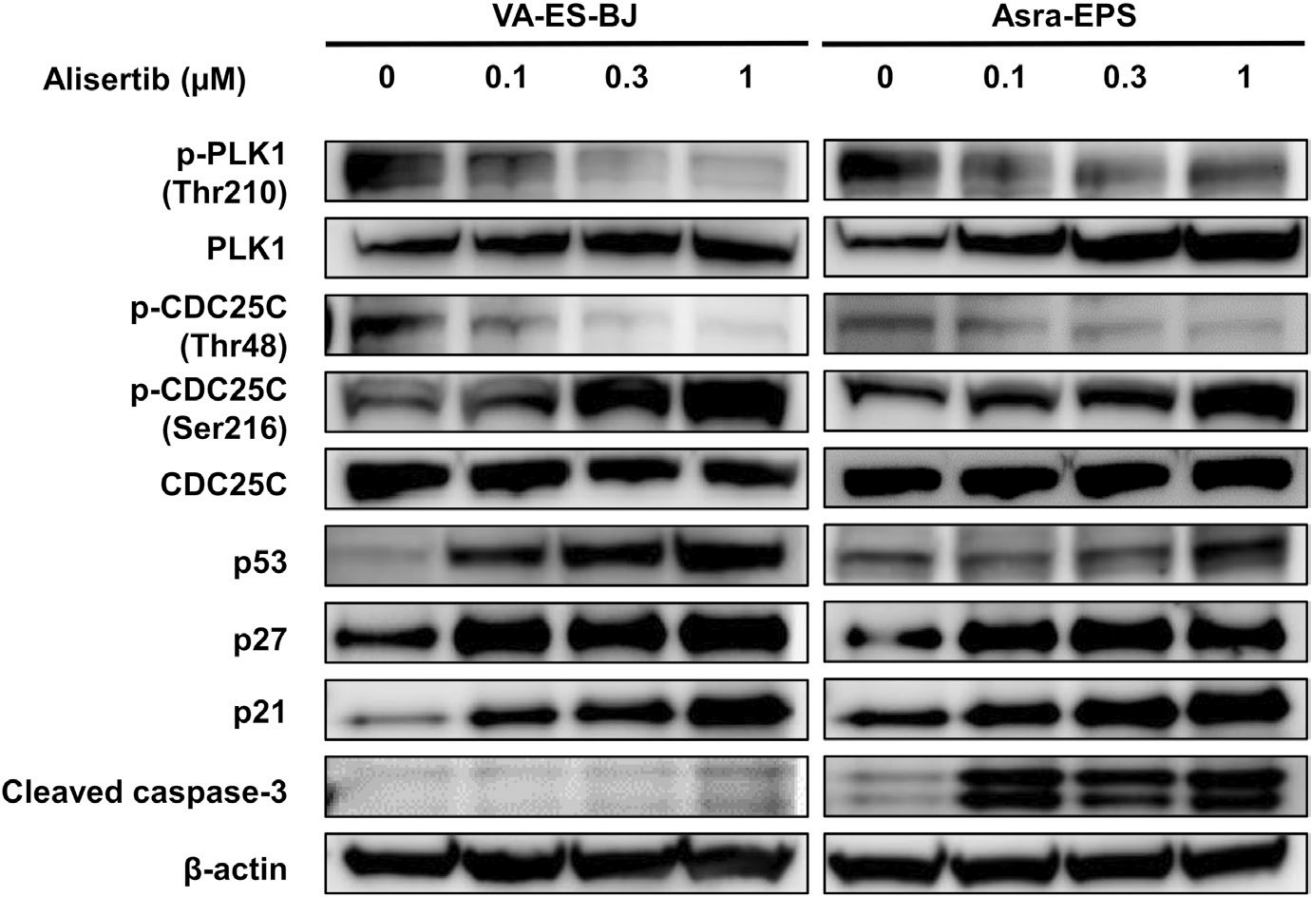
Alisertib downregulated the AURKA/PLK1/CDC25C axis and increased senescence and apoptosis markers in EpS. Effects of alisertib on AURKA‐related proteins. EpS cells were treated with 0.1–1 μM of alisertib for 24 h.

Next, we investigated which signaling pathway was involved in the alisertib‐induced cellular senescence and apoptosis. Notably, we found that AURKA inhibition with alisertib decreased the protein levels of p‐PLK1 (Thr210) and p‐CDC25C (Thr48), and increased p‐CDC25C (Ser216) levels in VA‐ES‐BJ and Asra‐EPS cell lines (Figure 5). Furthermore, alisertib upregulated the expression levels of p53, p27, p21, and cleaved caspase‐3, a major effector of apoptosis, in both EpS cell lines (Figure 5). These results suggest that alisertib induced cellular senescence and apoptosis by inhibiting AURKA/PLK1/CDC25C signaling in EpS cells.

### 3.6 Alisertib Abrogated EpS Tumor Growth by Inhibiting AURKA/PLK1/CDC25C Signaling In Vivo

**FIGURE 6:**
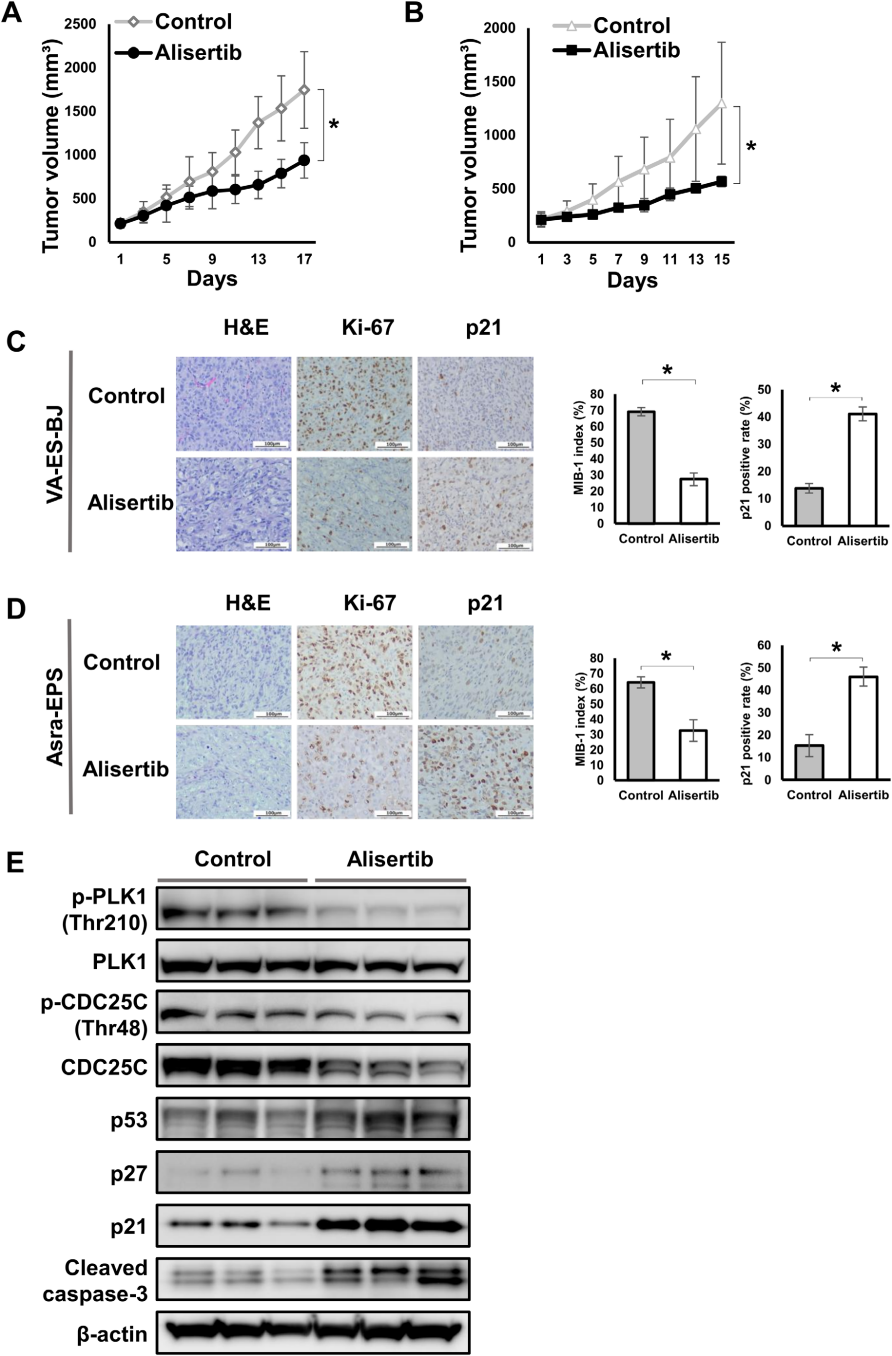
Alisertib decreased EpS xenograft tumor growth *in vivo*. (A) Effects of alisertib on VA‐ES‐BJ xenograft tumor volume. Points, mean; bars, SD. *p < 0.01. (B) Effects of alisertib on Asra‐EPS xenograft tumor volume. Points, mean; bars, SD. *p < 0.01. (C) Hematoxylin and eosin and immunohistochemical staining of Ki‐67 and p21 in both groups on VA‐ES‐BJ xenograft. Scale bars, 100 μm. Columns, mean; bars, SD. *p < 0.01. (D) Hematoxylin and eosin and immunohistochemical staining of Ki‐67 and p21 in both groups on Asra‐EPS xenograft. Scale bars, 100 μm. Columns, mean; bars, SD. *p < 0.01. (E) Effects of alisertib on AURKA‐related proteins in VA‐ES‐BJ xenograft tumors.

Finally, we examined the antitumor efficacy of alisertib on VA‐ES‐BJ and Asra‐EPS xenografted tumors in nude mice. Our results revealed that treatment with 30 mg/kg of alisertib significantly inhibited the growth of EpS tumor xenografts when compared with vehicle control (Figure 6A,B), with similar results having been obtained with respect to tumor weight (Figure S5A–D). Daily alisertib administration at 30 mg/kg did not appear to cause toxicity in mice based on body weight changes (Figure S5E).

Hematoxylin and eosin staining revealed lower cell population density in alisertib‐treated tumors than in vehicle‐treated tumors (Figure 6C,D). Immunostaining with cell proliferation marker Ki‐67 showed that compared with the control group, the alisertib‐treated group had a lower ratio of cells positive for Ki‐67, known as the MIB‐1 index, and a higher ratio of cells expressing p21 (Figure 6C,D).

Western blotting analyses were performed to evaluate the effects of alisertib treatment on AURKA‐related protein expression of VA‐ES‐BJ xenograft tumors. Similar to the in vitro results, alisertib‐treated mice showed inactivation of PLK1 and CDC25C and increased expression levels of p53, p27, p21, and cleaved caspase‐3 compared to vehicle‐treated mice (Figure 6E). These results demonstrate that alisertib also inhibited tumor growth and induced senescence and apoptosis of EpS xenograft tumors by inhibiting the AURKA/PLK1/CDC25C axis in vivo.

## 4 Discussion

INI1 is a subunit of the SWI/SNF ATP‐dependent chromatin remodeling complex in mammals [9]. The SWI/SNF complex mediates diverse biological pathways through epigenetic regulation of gene expression [7]. In fact, studies have estimated that over 20% of all cancers involve alterations in SWI/SNF subunits [31]. Considering that INI1 protein plays a critical role in epigenetic regulation, cell cycle progression, and crosstalk between various signaling cascades, loss of INI1 function has been found to upregulate several oncogenic signaling pathways associated with tumor proliferation and progression [18, 32, 33, 34, 35, 36, 37, 38]. INI1 loss, which was initially identified in MRT, has also been found with high frequency in EpS [8, 9, 10, 11, 12, 13]. Brenca et al. demonstrated that loss of INI1 expression in VA‐ES‐BJ was attributed to the homozygous deletion of *INI1* [39]. The present study found that the reintroduction of INI1 into VA‐ES‐BJ cells decreased the proliferation of xenografted tumors and abolished their capacity for forming colonies, indicating that INI1 loss plays an important role in the cell proliferation and tumorigenicity of EpS. Given our findings regarding INI1 reintroduction in EpS, it is intuitively appealing to consider INI1 reintroduction as the optimal therapeutic approach for EpS. Nonetheless, practical application remains exceedingly challenging considering the prevalence of *INI1* deletion in most EpS cases [10, 11, 12, 13]. Hence, targeting the downstream effector of INI1 should be a more practical treatment approach for EpS.

Loss of INI1 upregulates the expression of enhancer of zeste homolog 2 (EZH2) [35], which is one of the components of the catalytic subunit of the polycomb repressive complex 2 that catalyzes methylation of lysine 27 in histone H3 trimethylation, consequently repressing gene expression [40]. Studies have shown that EZH2 depletion and inhibition suppress tumorigenesis in INI1‐deficient cells, suggesting that EZH2 inhibition is a promising strategy for antitumor therapy [41, 42]. In 2020, the Food and Drug Administration approved an EZH2 inhibitor tazemetostat for the treatment of advanced EpS. Nonetheless, only 15% of patients responded to tazemetostat, with rapid development of resistance having been observed [43]. Furthermore, recent studies have highlighted other oncogenic signaling pathways that are derepressed in the setting of INI1 deficiency, including c‐Myc [33]. Indeed, the current study also observed that the reintroduction of INI1 downregulated EZH2 and c‐Myc in VA‐ES‐BJ cells (Figure S6A). However, our findings demonstrated that tazemetostat had limited antiproliferative effects in EpS (Figure S6B). Moreover, we had previously reported that other epigenetic inhibitors, namely histone deacetylase and bromodomain and extra‐terminal inhibitors, showed significant antitumor effects in clear cell sarcoma and synovial sarcoma, respectively [44, 45]. However, both inhibitors demonstrated modest effects in EpS.

AURKA has been garnering increasing recognition as a viable target for cancer therapy given its elevated expression across various malignancies [15, 16]. Additionally, Yamada et al. have shown that in clear cell sarcoma, the expression of the *EWS‐ATF1* fusion gene led to increased levels of AURKA and PLK1 [46]. Conversely, the presence of AURKA is not universally essential in all cell types, as its inhibition in normal cells such as NHDF has little impact on cellular proliferation. In fact, Lee et al. previously reported that INI1 was associated with the *AURKA* promoter and repressed *AURKA* transcription in RT cells, thereby promoting AURKA overexpression in INI1‐deficient RT cells [18]. AT‐rich interaction domain 1A, a component of the SWI/SNF chromatin remodeling complex, had also been found to occupy the *AURKA* gene promoter and negatively regulate its transcription [47]. However, no study has yet investigated the association between INI1 deficiency and AURKA expression in EpS. Similar to findings in other INI1‐deficient malignancies, our results showed that INI1 loss derepressed AURKA expression in EpS cell lines. However, shRNA‐induced downregulation of *INI1* had no effect on AURKA expression and sensitivity to alisertib in NHDF cells (Figure S7A–C, Table S4, Data S1). INI1 is known to function as a chromatin remodeler and exert repressive effects directly on the promoter region of AURKA [18, 48, 49]. To address the discrepancies in AURKA expression observed between EpS and NHDF under conditions of INI1 presence or absence, CUT&RUN analysis was conducted, focusing on the *AURKA* promoter region (Data S1). The CUT&RUN qPCR assay demonstrated that INI1 deficiency in EpS correlated with increased levels of H3K27ac, H3K9ac, and H3K4me3 at the *AURKA* promoter, along with the release of INI1‐mediated direct repression (Figure S8A–E). In contrast, no such relationship was detected in NHDF (Figure S8A–E). These results suggest that the regulatory influence of INI1 on AURKA expression is cell type‐specific and that INI1 loss in INI1‐deficient tumors should drive AURKA overexpression via chromatin structural alterations and the abrogation of INI1‐mediated repression. INI1 directly governs and controls AURKA expression in tumor cells; however, in INI1‐deficient tumors such as EpS, the loss of INI1 leads to increased AURKA expression, creating an environment in which tumor cells become dependent on AURKA. As a result, inhibiting AURKA may yield more pronounced therapeutic effects. In contrast, in other tumors or normal cells where INI1 is present and AURKA expression is either properly regulated or controlled by other factors, the therapeutic efficacy remains limited. This finding suggests that AURKA is a downstream target of INI1 in EpS but not NHDF, indicating that AURKA regulation is dependent on cell type.

AURKA is involved in PLK1 phosphorylation, thereby promoting mitosis at the G2/M phase. PLK1 promotes the activation of CDC25C, which is an activator of cyclin B1 and CDK1, and subsequently triggers mitotic entry. Through microarray‐based gene expression profiling, Morozof et al. discovered that INI1 also repressed the mitotic gene *PLK1* in RT, subsequently promoting PLK1 overexpression in INI1‐deficient RT cells [50]. We had previously revealed through transcriptome analysis that *AURKA*, *PLK1*, and *CDC25C* were upregulated in EpS organoid‐derived xenograft compared to normal tissue [51]. In the mentioned study, the reintroduction of INI1 into VA‐ES‐BJ cells reduced the expression of AURKA and its downstream effectors PLK1 and CDC25C. Furthermore, *AURKA* knockdown inhibited PLK1 and CDC25C activation in EpS cell lines. These results demonstrate that the reduction in the expression of PLK1 and CDC25C can be attributed to INI1 deficiency and that their persistent activation is mediated by AURKA.

Several completed or ongoing phase I to III clinical trials investigating the effects of alisertib, a selective, small‐molecule AURKA inhibitor, in advanced solid tumors and hematologic malignancies have shown some promising results [19, 20, 21, 22, 23, 24, 25]. To date, however, evidence regarding the effects of pharmacological AURKA inhibition on EpS has been lacking. We initially hypothesized that alisertib would exert antitumor effects against EpS by inhibiting AURKA/PLK1/CDC25C signaling. Accordingly, the current study found that alisertib markedly suppressed cell growth, induced cell cycle arrest in the G2/M phase, and promoted senescence and apoptosis by downmodulating the AURKA/PLK1/CDC25C axis in INI1‐deficient EpS cells. Interestingly, we found that the reintroduction of INI1 mitigated the impact of alisertib in VA‐ES‐BJ cells, suggesting that INI1 deletion results in higher sensitivity to alisertib in EpS. These results demonstrate that EpS cells are reliant on the AURKA/PLK1/CDC25C axis for their aberrant proliferation and survival. Hence, AURKA inhibition should play an important role in the management of patients with EpS, warranting further evaluation in clinical studies.

Other studies have shown that INI1 transcriptionally activates p16 and represses cyclin D1 in MRT and AT/RT [36, 37]. Jamshidi and colleagues demonstrated significantly reduced immunostaining for p16 in 6 of the 16 EpS samples analyzed [52]. Moreover, they found homozygous deletion of the *CDKN2A* locus, which encodes p16 and p14, in EpS cell lines VA‐ES‐BJ and HS‐ES. In the present study, p16 expression was not detected in both EpS cell lines (data not shown). Furthermore, the reintroduction of INI1 in VA‐ES‐BJ did not enhance p16 expression unlike MRT and AT/RT (Figure S6A). Loss of p16 expression occurred independently of INI1 deficiency in EpS, suggesting that oncogenic mechanisms in EpS differ from those in MRT and AT/RT. Our study demonstrated that reintroduction of INI1 in VA‐ES‐BJ cells promoted an increase in p21 and p27 and a decrease in cyclin B1. AURKA inhibition with alisertib upregulated the expression of p27 and p21 by inactivating the AURKA/PLK1/CDC25C axis in EpS cells, similar to that observed with the reintroduction of INI1.

While these are useful findings, they are limited by the cell line‐based study. New clinical trials adapted to the INI1 negative tumors, including EpS, would help to further clarify the efficacy.

In conclusion, the current study found that loss of INI1 expression promoted proliferation and tumorigenesis in EpS. Moreover, INI1 deficiency in EpS induced persistent activation of the AURKA/PLK1/CDC25C axis. We also found that inhibition of AURKA with alisertib remarkably repressed cell proliferation and induced G2/M cell cycle arrest, cellular senescence, and apoptosis in EpS. Hence, targeting the AURKA/PLK1/CDC25C axis has emerged as a promising therapeutic strategy for treating EpS displaying marked resistance to conventional chemotherapy regimens.

## Author Contributions

**Akitomo Inoue:** conceptualization, data curation, formal analysis, investigation, methodology, resources, writing – original draft. **Hidetatsu Outani:** conceptualization, funding acquisition, methodology, project administration, validation. **Yoshinori Imura:** conceptualization, funding acquisition, investigation, methodology, project administration, supervision, validation, writing – original draft, writing – review and editing. **Sho Nakai:** conceptualization, funding acquisition, investigation, methodology, project administration. **Haruna Takami:** data curation, formal analysis, resources. **Yuki Kotani:** data curation, formal analysis, resources. **Hirokazu Mae:** formal analysis, resources. **Seiji Okada:** conceptualization, funding acquisition, methodology, project administration, supervision, writing – review and editing.

## Ethics Statement

Approval of the research protocol by an Institutional Reviewer Board: This study was conducted in accordance with the Declaration of Helsinki. Ethical approval for this study was obtained from the Institutional Review Board of the Ethics Committee of Osaka University Graduate School of Medicine (approval number: 11044–4). Informed Consent: N/A. Registry and the Registration No. of the study/trial: N/A. Animal Studies: Animal studies were approved by the Institutional Review Board of Osaka University Animal Experiments Committee (approval number: 03–000‐002).

## Conflicts of Interest

The authors declare no conflicts of interest.

## Supporting information


Figure S1.

Figure S2.

Figure S3.

Figure S4.

Figure S5.

Figure S6.

Figure S7.

Figure S8.

Table S1.
**Table S2**.
**Table S3**.
**Table S4**.

Data S1.

## Data Availability

The authors confirm that the data supporting the findings of this study are available within the article and its supporting materials.
