# Supplementary figures and images for: AURKA/PLK1/CDC25C Axis as a Novel Therapeutic Target in INI1‐Deficient Epithelioid Sarcoma
Source: Cancer Sci. 2025 Jan 9;116(4):976–89. doi: 10.1111/cas.16438 (PMC11967267; doi:10.1111/cas.16438)

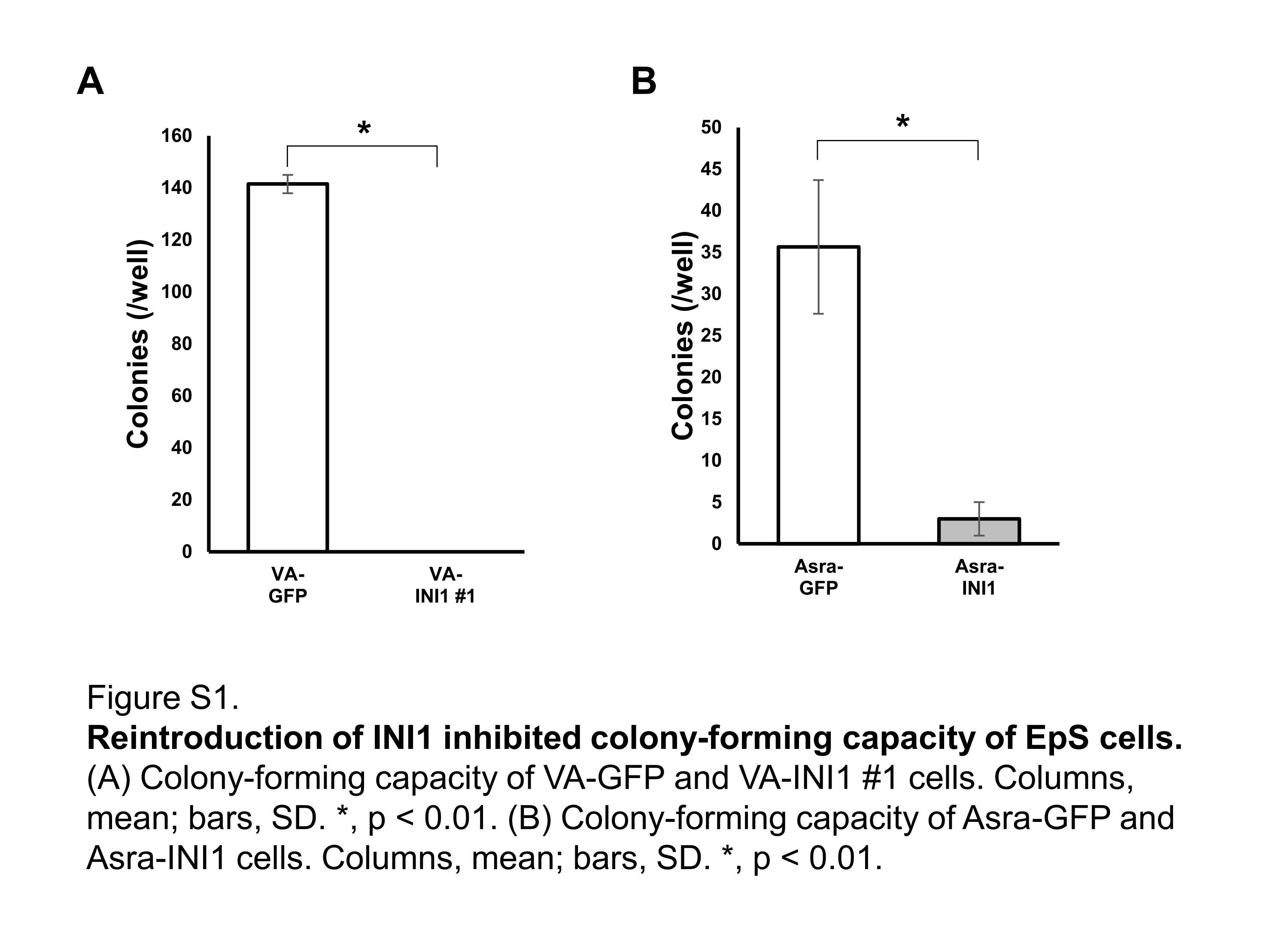

Supplement: Supplementary file 1 — Figure S1. [file CAS-116-976-s003.tif]

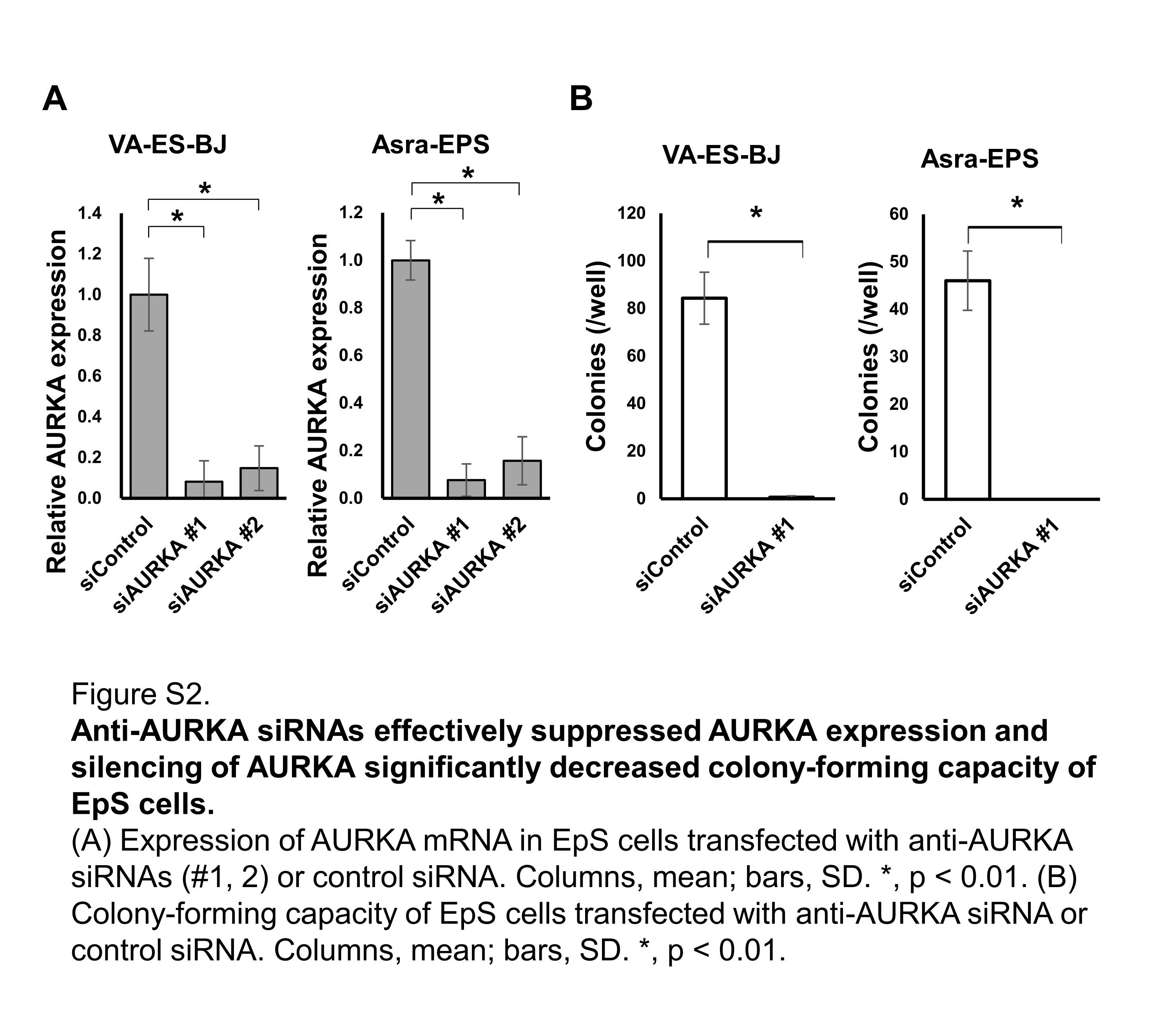

Supplement: Supplementary file 2 — Figure S2. [file CAS-116-976-s004.tif]

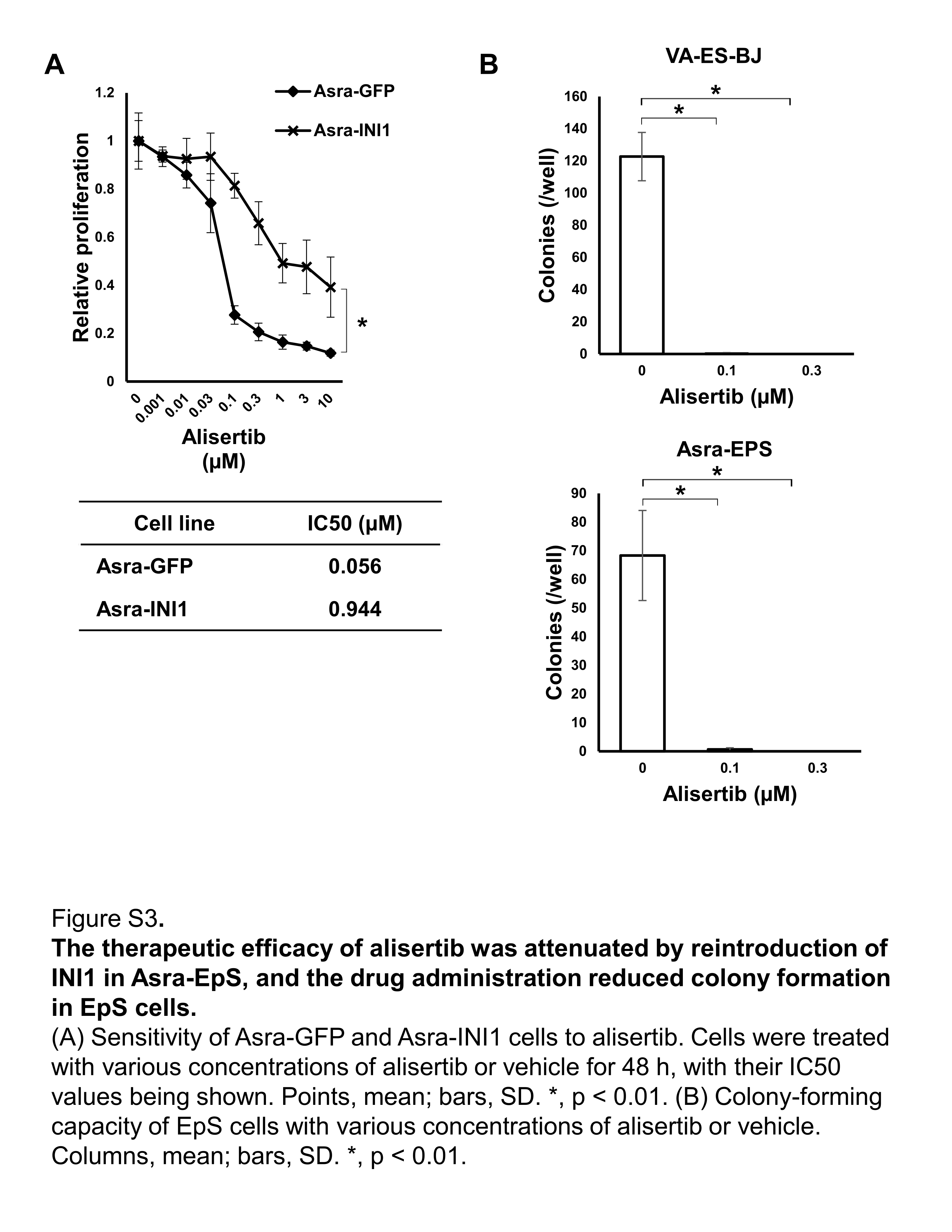

Supplement: Supplementary file 3 — Figure S3. [file CAS-116-976-s006.tif]

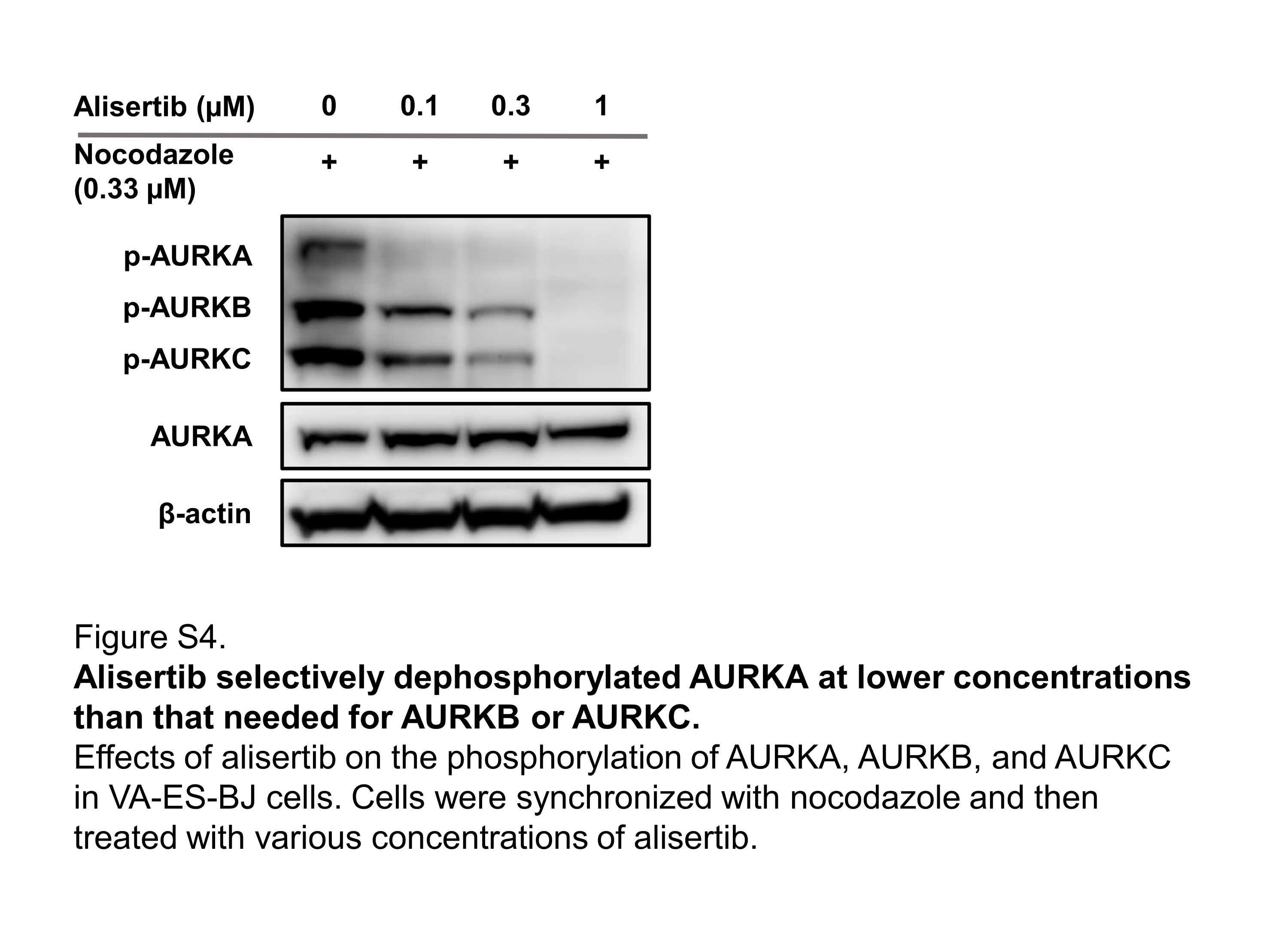

Supplement: Supplementary file 4 — Figure S4. [file CAS-116-976-s008.tif]

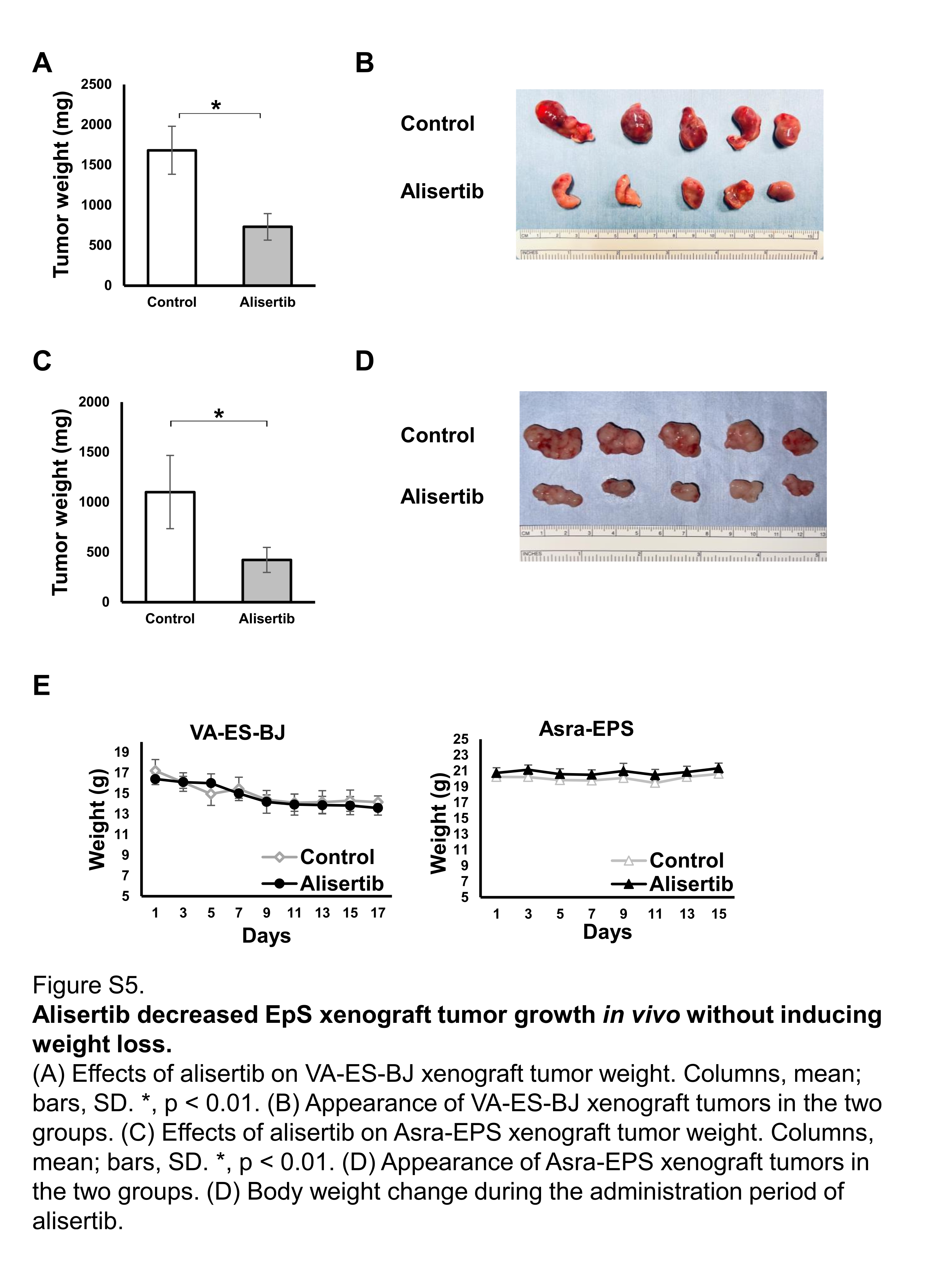

Supplement: Supplementary file 5 — Figure S5. [file CAS-116-976-s001.tif]

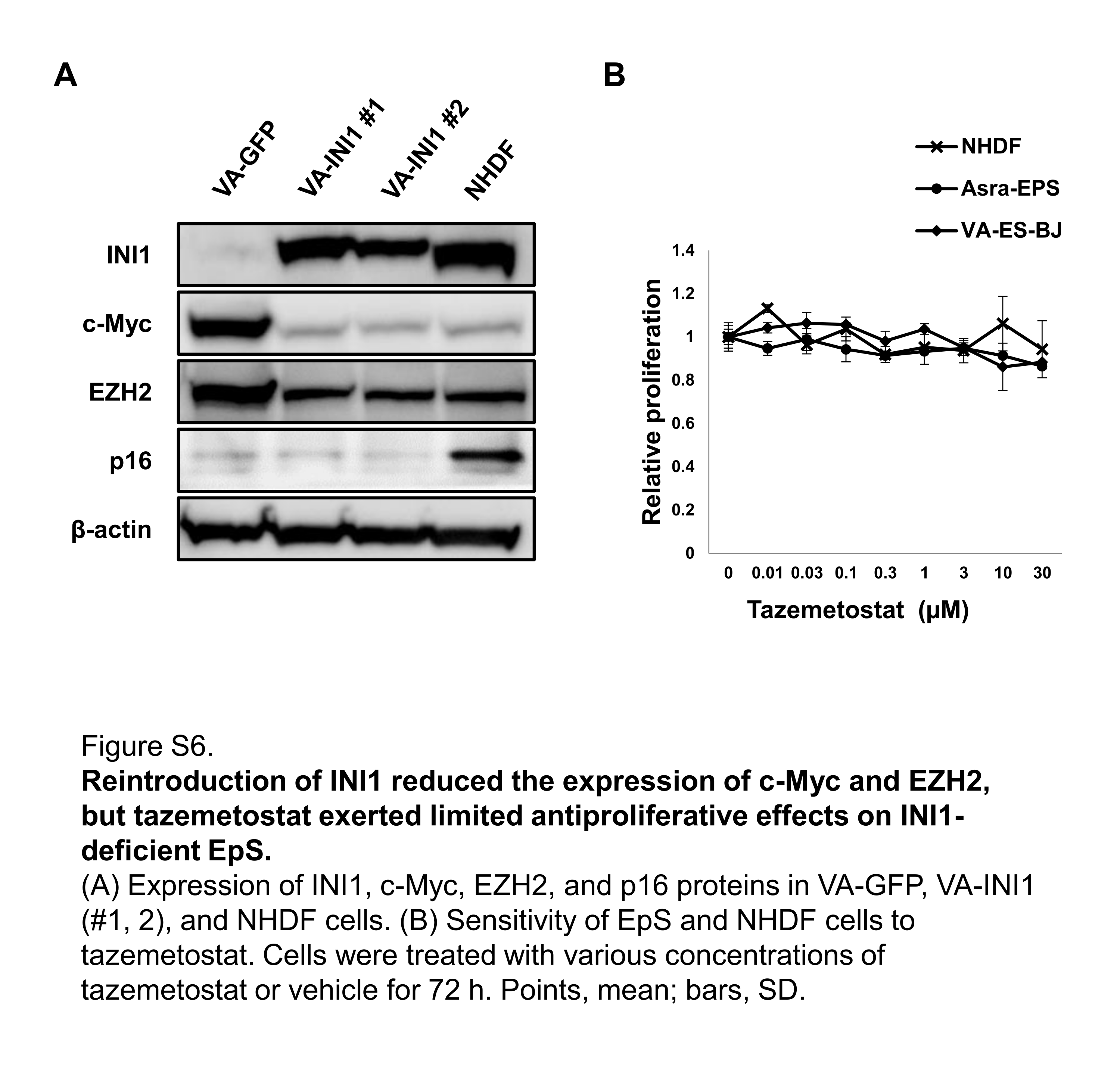

Supplement: Supplementary file 6 — Figure S6. [file CAS-116-976-s009.tif]

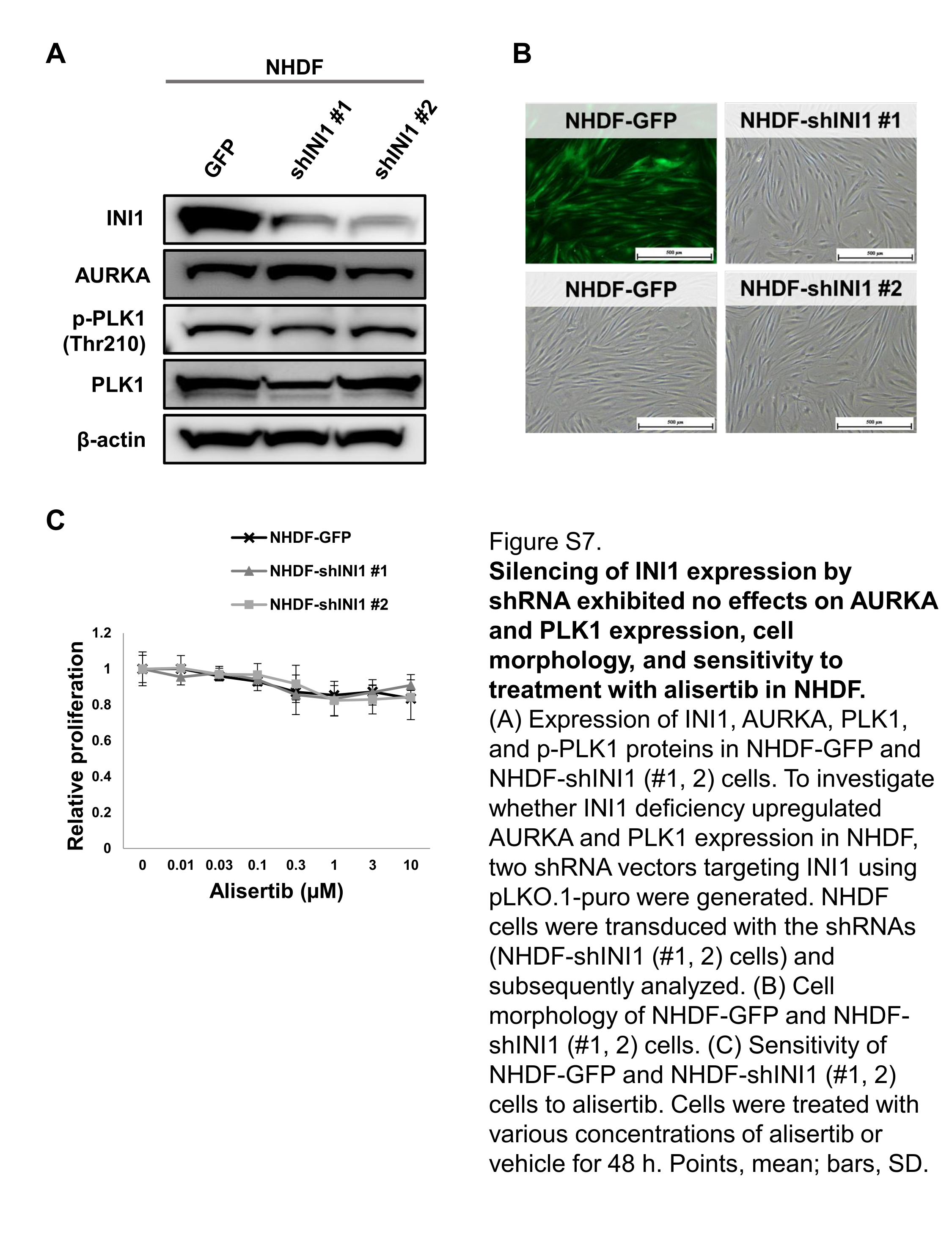

Supplement: Supplementary file 7 — Figure S7. [file CAS-116-976-s007.tif]

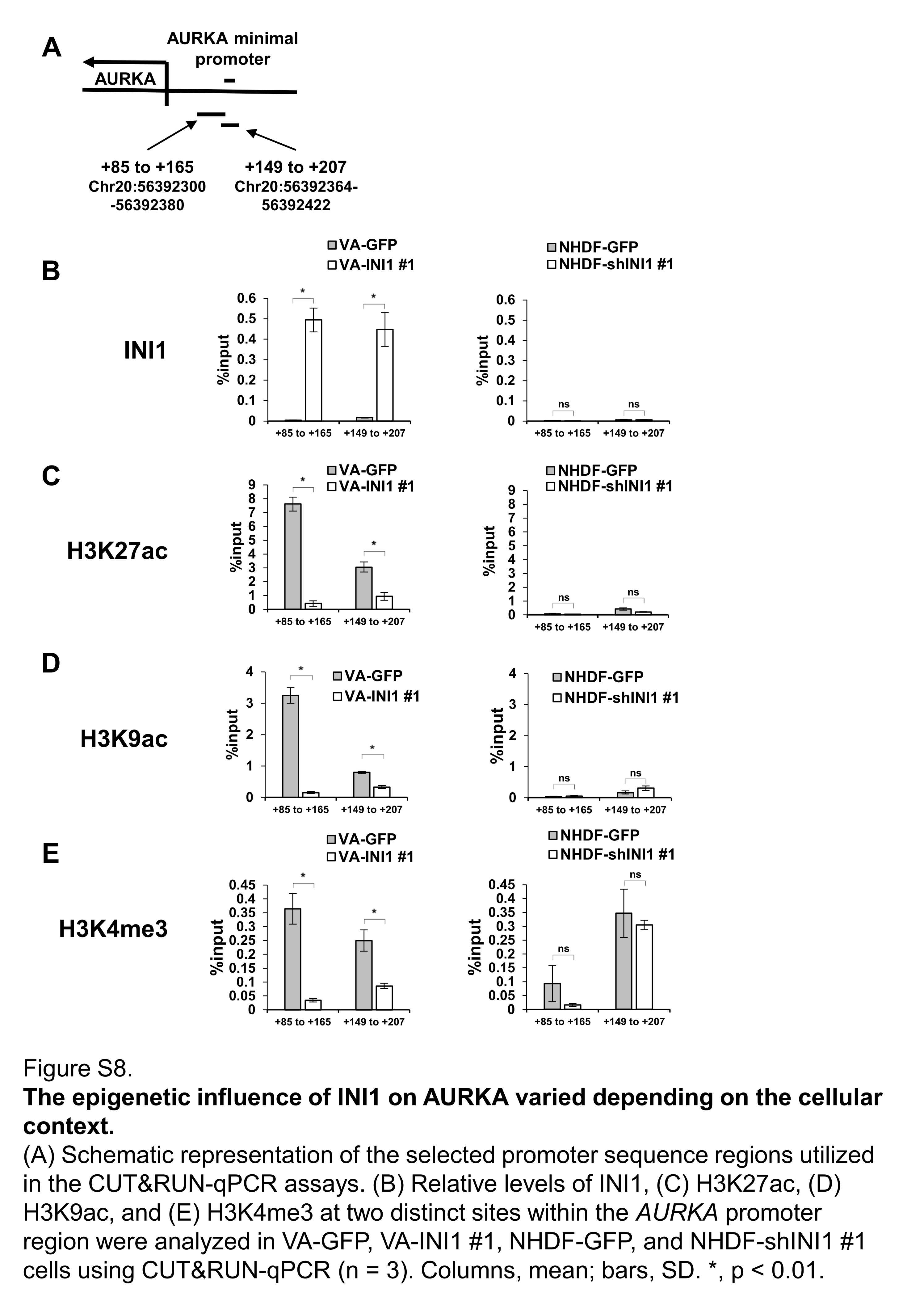

Supplement: Supplementary file 8 — Figure S8. [file CAS-116-976-s010.tif]
