# Supplementary material for: AURKA/PLK1/CDC25C Axis as a Novel Therapeutic Target in INI1‐Deficient Epithelioid Sarcoma
Source: Cancer Sci. 2025 Jan 9;116(4):976–89. doi: 10.1111/cas.16438 (PMC11967267; doi:10.1111/cas.16438)
Supplement: Supplementary file 10 — Data S1. [file CAS-116-976-s002.docx]

**Supplementary Methods Data S1**

**Lenti viral-mediated forced expression of GFP and INI1**

To clone human INI1 in lentivirus expression vectors, Gateway technology was used (Invitrogen). To force the expression of INI1, PCR products with attB sequences at both ends of the coding region of the full-length coding sequence of normal human INI1 gene were amplified by RT-PCR. These sequences were then cloned into the pDONR221 vector (Invitrogen) for BP recombination reaction. pLenti6.4/R4R2/V5-DEST was then added to this reaction solution, and LR recombination reaction was performed to recombine the resulting plasmid with INI1. Transformation was performed using DH5α cells, and all positive clones were verified via nucleotide sequence analysis. On the day prior to transduction, 293FT cells were seeded at a density of 4 × 10⁶ cells per 100-mm dish. The next day, INI1 lentiviral plasmids with ViraPower Packaging Mix (pLP1, pLP2, and pLP/VSVG) were co-transfected to 293FT cells with Lipofectamine 2000 (Invitrogen) according to the manufacturer's recommendations and replated in 10 mL of fresh medium after 24 h. Separately, VA-ES-BJ cells were seeded at a density of 8 × 10⁵ cells per 100-mm dish. After 24 h of incubation, virus-containing supernatants from these 293FT cultures were filtered through a 0.45-μm cellulose acetate filter (Schleicher & Schuell) and supplemented with 4 μg/mL polybrene (Nacalai Tesque). VA-ES-BJ cells were transduced with the lentiviral particles for 4 h to overnight, after which the INI1-overexpressing clones were selected using 10 µg/mL Blasticidin S. Single clonal selection was performed and harvested for 2 to 3 months. Using the same method, enhanced GFP was transduced as a control. INI1 overexpression was verified via RT-qPCR and western blotting. For Asra-EPS, gene transduction was performed using the same method; however, since no proliferation was observed in single cells and single-cell cloning was not feasible, experiments were conducted following antibiotic selection.

**shRNA-mediated INI1 silencing**

For gene silencing, shRNA constructs targeting INI1 were designed and cloned into the pLKO.1-puro vector (Addgene), with pLKO.1-EGFP-puro serving as a negative control. The shRNA target sequences for INI1 were listed in Table S4. The transduction process was performed as previously described. Stably transduced cells were selected using 1 μg/mL puromycin for 5 days. The efficiency of INI1 knockdown was validated through RT-qPCR and Western blot analysis.

**Promoter interaction analysis via CUT&RUN-qPCR**

To analyze promoter interactions, Cleavage Under Targets and Release Using Nuclease (CUT&RUN) assays were conducted utilizing the CUT&RUN Assay Kit (Cell Signaling Technology) in accordance with the manufacturer’s protocol. Cells were seeded at a density of 1 × 10⁶ cells per 6-cm dish and cultured for 24 h. For each reaction, 1 × 10⁵ cells were used, immobilized on concanavalin A beads, and permeabilized with a digitonin-containing buffer. Antibodies (listed in Table S1) were applied at a 1:50 dilution and incubated overnight at 4°C. Antibody-bound DNA fragments were subsequently purified using DNA purification buffers and spin columns (Cell Signaling Technology).

The target DNA regions were detected via SYBR Green RT-qPCR using primers specific to the loci enriched by the antibodies on the human *AURKA* promoter, as determined by prior studies and ChIP-Atlas database (https://chip-atlas.org). The forward and reverse primer sequences used for CUT&RUN-qPCR are provided in Table S2. Gene expression analysis was performed using the StepOnePlus Real-Time PCR System (Applied Biosystems).
